# Influence of voluntary isocapnic hyperpnoea on recovery after high-intensity exercise in elite short-track speedskaters – randomized controlled trial

**DOI:** 10.1186/s13102-024-00927-0

**Published:** 2024-06-20

**Authors:** Tomasz Kowalski, Adrian Wilk, Kinga Rębiś, Kim-Morgaine Lohse, Dorota Sadowska, Andrzej Klusiewicz

**Affiliations:** 1 Department of Physiology, Institute of Sport—National Research Institute, Warsaw, Poland; 2 Centre for Digital Health Interventions, Department of Management, Technology, and Economics, ETH Zurich, Zurich, Switzerland; 3 Faculty of Physical Education and Sport in Biala Podlaska, Department of Physiology and Biochemistry, Józef Piłsudski University of Physical Education in Warsaw, Biala Podlaska, Poland

**Keywords:** Respiratory muscle training, Voluntary isocapnic hyperpnoea, Recovery, Breathing, Speedskating

## Abstract

Respiratory muscle training plays a significant role in reducing blood lactate concentration (bLa) and attenuating negative physiological stress reactions. Therefore, we investigated if voluntary isocapnic hyperpnoea (VIH) performed after a maximum anaerobic effort influences bLa and perceived fatigue level in well-trained speedskaters. 39 elite short-track speedskaters participated in a trial with two parallel groups: experimental and control. All the participants performed the Wingate Anaerobic Test (WAnT). The experimental group performed a VIH-based recovery protocol 20 min after exercise, the control group used passive recovery only. Blood samples were taken 3 and 30 min after the WAnT to measure bLa. Fatigue was self-appraised on a 0–10 perceived rating-of-fatigue (ROF) scale 3 and 30 min after the WAnT. Noteworthy, but not statistically significant changes between the experimental and control groups were observed for changes in bLa (*p* = 0.101). However, statistically significant changes between the groups were found for ROF (*p* = 0.003, η_p_^2^ = 0.211, ω^2^ = 0.106). Moreover, statistically significant interactions between post-exercise bLa clearance and VO_2_max (*p* = 0.028) and inspiratory muscle strength (*p* = 0.040) were observed. Our findings provided preliminary insight that VIH may be an efficient recovery protocol after anaerobic exercise performed by elite athletes. The association between VO_2_max and post-exercise bLa clearance indicates the vital role of aerobic fitness in repeated-efforts ability in short-track speedskaters. The study was registered at ClinicalTrials.gov as NCT05994092 on 15th August 2023.

**Supplementary Information:**

The online version contains supplementary material available at 10.1186/s13102-024-00927-0.

## Introduction

Based on literature reviews, it has been identified that respiratory muscle training (RMT) has the potential to enhance performance in various scenarios, such as time trials, intermittent incremental tests, and constant load tests [1, 2]. Additionally, RMT has shown promise in improving respiratory muscle endurance and strength [1–4], reducing perceived exertion or breathlessness [1], and alleviating respiratory fatigue during exercise in both normoxia and hypoxia [1, 5]. More recently, alternative applications of RMT were presented, for example as an efficient warm-up method [6]. Interestingly, Brown et al. suggested that respiratory muscles play a significant role in reducing blood lactate concentration (bLa) [7], which was confirmed in the systematic review and meta-analysis prepared by Illi et al. [2]. Therefore, we speculated that RMT may be applied as an active recovery protocol by modifying post-exercise lactate accumulation and clearance.

Multiple studies have demonstrated that active recovery may offer performance benefits during intermittent exercise when compared to passive recovery [8–10]. More specifically, bLa accumulation has been associated with physical fatigue [11], whereas a fast decrease in bLa has been associated with optimal performance during repeated exercise bouts [12, 13]. The significant link between changes in bLa after RMT and improvements in repeated performance was established by Romer et al. [14]. Their findings revealed that alterations in lactate concentration accounted for up to 52% of the variation in performance.

The specific mechanisms responsible for the bLa decrease associated with RMT are not fully understood. Possible explanations include enhanced oxidative capacity and lactate uptake, as well as improved lactate transport capacity of the trained respiratory muscles [7, 15]. Animal studies revealed that respiratory muscles are ‘lactate netto consumers’, since during exercise increased intra-muscle lactate concentration in the absence of glycogen utilization was observed, indicating increased lactate uptake rather than production [16]. It was also reported that approximately half of the energy required by the diaphragm came from carbohydrate metabolism, mostly in the form of lactate utilization [17]. Moreover, the diaphragm activity may attenuate negative physiological stress reactions due to vagal nerve stimulation [18, 19]. Consequently, via autonomic nervous system modulation, the perceived rating-of-fatigue (ROF) and associated rating of perceived exertion (RPE) may decrease, positively influencing subsequent performance [20, 21].

Using RMT techniques as a recovery tool may be exceptionally useful in sports that do not allow for well-tested, traditional recovery protocols such as cycling- or running-based active recovery, thermal interventions, or advanced physiotherapy protocols, yet still require repeated high-intensity efforts during the competition. Short-track speedskating, a dynamic winter olympic sport, is a representative example of such a situation. Typically, short-track competition involves multiple races per day, highlighting the demanding nature of repeated performances and the critical role of recovery between races. However, the time interval between races may be as little as 20–30 min, which severely limits the available recovery protocols. Moreover, due to the high technical demands of ice skating, any deviation from optimal muscle tension and neuromuscular coordination may lead to a deterioration of performance [22]. Therefore, implementing recovery protocols that do not engage skeletal limb muscles may be preferred. As resistance-based RMT methods induce significant homeostasis disturbances and cause accompanying symptoms like headaches or dizziness, we investigated another method, known as voluntary isocapnic hyperpnea (VIH) [23]. The available literature about the impact of post-exercise VIH is scarce and remains inconclusive [15, 24–26].

We hypothesized that RMT could serve as a recovery protocol by reducing acute bLa and diminishing ROF through a single RMT-based effort. Therefore, we investigated the influence of the low-intensity VIH protocol performed after a maximum anaerobic effort in well-trained short-track speedskaters.

## Materials and methods

The study design was reviewed and approved by the Institute of Sport - National Research Institute Ethics Committee (approval no KEBN-23-78-TK). All the procedures were carried out in agreement with the Declaration of Helsinki. Informed written consent was obtained from all study participants. CONSORT guidelines for reporting randomized trials were applied. The study was registered at ClinicalTrials.gov as NCT05994092 on 15th August 2023.

### Participants’ characteristics

39 short-track speedskaters (17 females, 22 males) completed the study. They were classified in Tier 4 or Tier 5 according to the Participant Classification Framework [27], as elite or world-class athletes. The criteria for study inclusion were: valid medical certificate to compete in speedskating, lack of previous experience with RMT, elite or world-class performance status, and at least 6 years of athletic training. The exclusion criteria were: any chronic medical condition, any acute medical condition within the last 3 months, and any ongoing medication intake. All the participants were recruited with convenience sampling among national teams from 3 different countries. The recruitment took place in May and June 2023. Of the 45 athletes initially recruited, 6 dropped out due to lack of formal eligibility or health constraints. Finally, 39 completed the study. All the participants were in the base training period, with 6 to 8 weeks of regular training after a post-season recovery period. The selected athletes followed similar training programs, as they worked with the same coaching group. The characteristics of participants who completed the study are presented in Table 1.

**Table 1:** Basic participants’ characteristics

Variable/Group	Experimental group (*n* = 21)	Control group (*n* = 18)
Age (years)	20.2 ± 2.7	22.0 ± 4.4
Body mass (kg)	65.1 ± 8.8	67.0 ± 10.7
Body height (cm)	173.0 ± 8.3	172.1 ± 7.8
VO_2_max (mL·kg⁻¹·min⁻¹)	53.5 ± 6.4	55.1 ± 5.5
S-Index Test score (cmH_2_O)	132.1 ± 26.7	138.7 ± 27.1
Maximum power (W·kg⁻¹)	12.9 ± 1.0	13.3 ± 1.6
Anaerobic capacity (J·kg⁻¹)	311.4 ± 28.3	316.6 ± 30.4

Values are mean ± standard deviation. No statistically significant differences in any parameter were found between the groups (*p* > 0.05).

### Study design and data collection

The study was conducted as a randomized controlled trial with two parallel groups: experimental and control. Stratified randomization to assign the participants to either the experimental or the control group was used by the authors of the study. First, the participants were assigned to subgroups based on their membership in either the National Development Team or the National Elite Team to account for the training status and age. Next, the participants were assigned to subgroups based on their sex. Then, inside the subgroups, the participants were assigned to either experimental or control group based on the coin toss. In the experimental group were 10 (47.62%) females and 11 (52.38%) males, while in the control group were 7 (38.89%) females and 11 (61.11%) males.

All the study participants performed the 30-second Wingate Anaerobic Test (WAnT) with maximal effort. The WAnT, conducted with Monark 874E Cycle Ergometer (Monark Exercise AB, Sweden), was used to measure maximum power output and anaerobic capacity. Both parameters were computed with dedicated software (MCE 6.0, JBA Z. Staniak, Poland) linked to the cycle ergometer. Prior to the WAnT, a standard warm-up of 5 min was performed with a load of 0.8–1.2 W/kg. Then, the participants performed a maximal 6-second sprint with a load adjusted to 7.5% of individual body mass. Following a 2-minute rest period, the athletes underwent the 30-second WAnT with the load adjusted to 7.5% of individual body mass. The objective for the subjects was to achieve the highest possible peak power as fast as possible and maintain the highest power output throughout the whole test duration. Loud and dynamic verbal encouragement was provided.

**Fig. 1:**
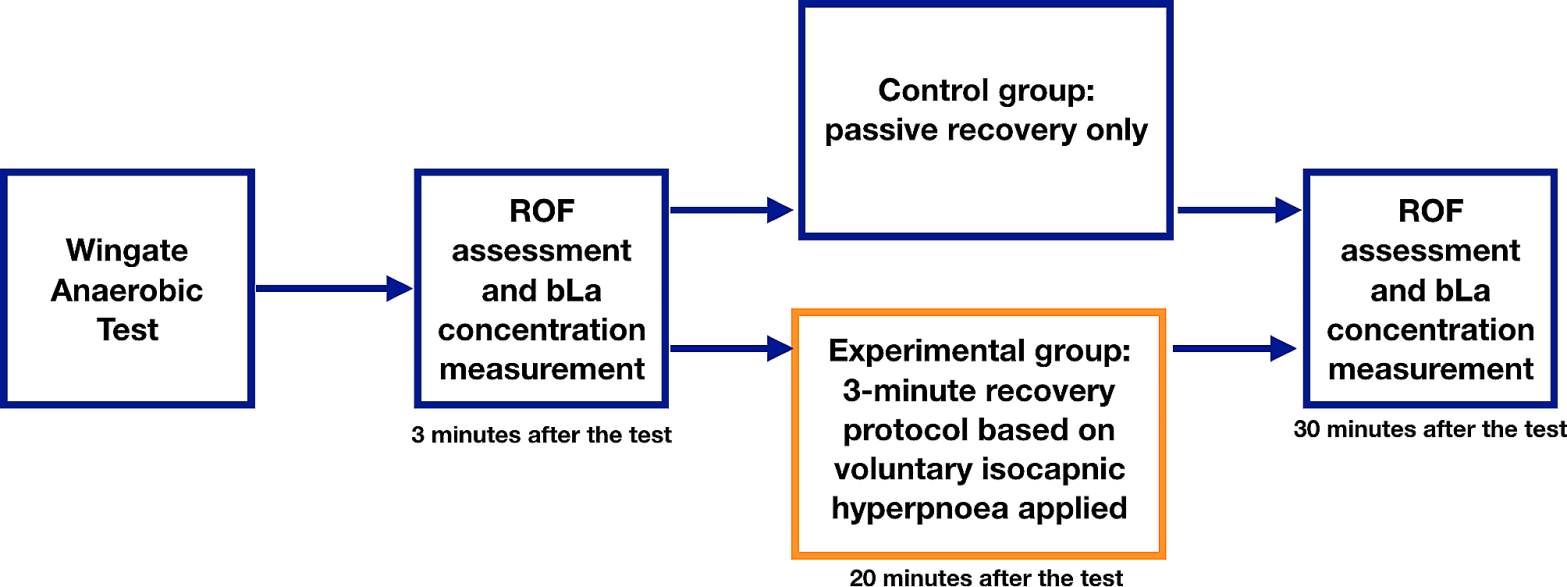
Testing design timeline. ROF - declared rating-of-fatigue, bLa - blood lactate

The experimental group performed a recovery protocol based on low-intensity VIH 20 min after exercise, whereas the control group did not perform any recovery protocol and used passive recovery only. The recovery protocol consisted of 3 min of purposeful and energetic breathing with 20 breaths·min-1 frequency. The participants were instructed to use diaphragmatic breathing patterns and minimize upper chest and shoulder movements. The Isocapnic BreathWayBetter devices (Isocapnic Technologies Inc, Kelowna, Canada) with 6-liter bags were used. The manufacturer’s app was used to provide visual guidance to the participants. The protocol was performed in a seating position, under the supervision of a qualified physiotherapist. All study participants were advised to sit during the 30-minute post-exercise period, with minimal walking to meet the physiological needs allowed. No drinking and eating were allowed. The blood samples were taken 3 min and 30 min after cessation of the exercise to measure the bLa. The ROF numerical scale (0–10) was presented to the participants 3 min and 30 min after cessation of the exercise to measure present ROF [28]. A visual presentation of the testing design timeline is presented in Fig. 1.

Body composition was assessed between 7:00 and 7:30 AM, prior to breakfast. The WAnT took place between 9:30 and 10:30 AM. Cardiopulmonary exercise testing was performed between 12:30 and 16:30. The S-Index Test took place on the next day in the morning. Body composition was assessed using a bioelectrical impedance analysis system (Tanita BC-420MA, Japan). The breath-by-breath cardiopulmonary exercise testing was performed with Cortex Metamax B3 (Cortex Biophysik GmbH, Leipzig, Germany) and Cyclus II Ergometer (RBM, Leipzig, Germany). All the participants completed an incremental ramp test to exhaustion starting from 55 to 70 W and gradually increasing the load by 0.17–0.28 W·sec-1. The load was individually adjusted based on body mass and previous test results. The highest average oxygen uptake for 15 s was defined as maximum oxygen uptake (VO_2_max). All the participants fulfilled the following criteria of maximum effort: (1) present oxygen uptake - work rate plateau, (2) declared exertion during CPET ≥ 18 in the Borg scale, (3) bLa ≥ 8 mmol·L⁻¹, (4) respiratory exchange ratio ≥ 1.10. Inspiratory muscle strength was assessed using S-Index Test performed with the POWERbreathe K5 device (POWERbreathe International Ltd., Southam, UK). The test was performed with 8 forceful and dynamic inspiratory maneuvers from residual volume to full inspiratory capacity, in a standing position, after a respiratory warm-up consisting of 10 inspiratory maneuvers [29].

Blood samples were taken from fingertips by skilled technicians in 20 uL capillary tubes. bLa was measured with the Super GL2 analyser (Dr. Müller Gerätebau GmbH, Freital, Germany). The researchers performing testing and the laboratory technicians performing biochemistry assays were kept blinded to the group allocation. The study participants were familiar with the testing procedures and numerical fatigue assessment, as they had used it in training and testing many times before. All the testing procedures were conducted at the Institute of Sport - National Research Institute (Warsaw, Poland) or its temporary field station (Gdansk, Poland). Study participants were required not to undertake demanding physical training or long-distance travel for 48 h before the testing.

### Statistical analysis

The normality of data distribution was assessed with the Shapiro-Wilk test and visual analysis of plot figures. Repeated measures analysis of variance (ANOVA) was applied to analyse the differences in bLa and ROF changes between the experimental and control groups. Additionally, homogeneity was assessed with Levene’s test. In significant main effects, post-hoc Bonferroni correction was used. The effect size was determined by partial eta squared (pη²) and omega squared (ω²) for significant relationships and was classified as small ≤ 0.06, moderate 0.07–0.14, or large > 0.15. Correlation and multiple regression analyses were conducted to examine bLa and ROF 30 min post-exercise. Significance was set at *p* < 0.05. All statistical analyses were performed using JASP statistical package (JASP Team, Amsterdam, Netherlands, Version 0.17.2).

## Results

Noteworthy, but not statistically significant changes between the experimental and control groups were observed for changes in bLa. However, statistically significant changes between the groups were observed for ROF.

There was no statistically significant difference in bLa changes between the groups (*p* = 0.101). During the monitored period, bLa decreased by 40.8% in the experimental group (14.5 ± 2.3 mmol·L⁻¹ at 3 min post-exercise and 8.6 ± 2.3 mmol·L⁻¹ at 30 min post-exercise) and 33.2% in the control group (15.3 ± 2.6 mmol·L⁻¹ at 3 min post-exercise and 10.2 ± 2.3 mmol·L⁻¹ at 30 min post-exercise).

There was a statistically significant difference in ROF changes between the groups (F(1, 37) = 9.098, *p* = 0.003, η_p_^2^ = 0.211, ω^2^ = 0.106). During the monitored period, ROF decreased by 63.7% in the experimental group (9.7 ± 0.5 at 3 min post-exercise and 3.5 ± 1.2 at 30 min post-exercise) and 46.5% in the control group (9.7 ± 0.6 at 3 min post-exercise and 5.2 ± 1.9 at 30 min post-exercise).

**Fig. 2:**
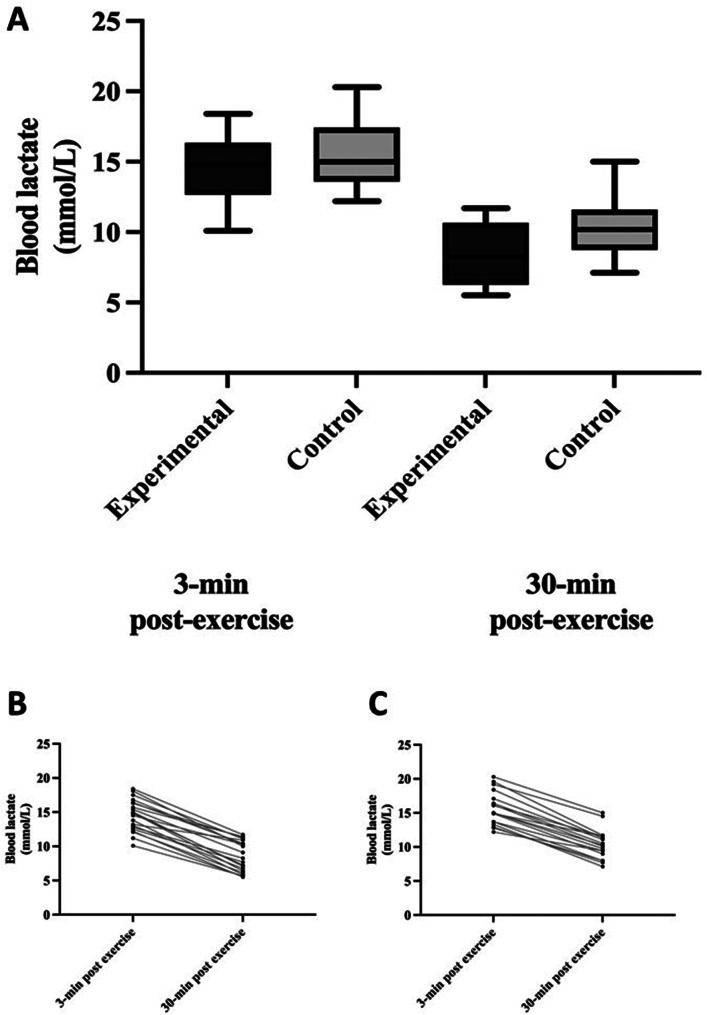
Panel A: The difference in bLa between measurements at 3 min and 30 min post-exercise for both experimental and control groups. Values are median and minimum or maximum values. Panel B: Individual bLa values for the experimental group. Panel C: Individual bLa values for the control group

**Fig. 3:**
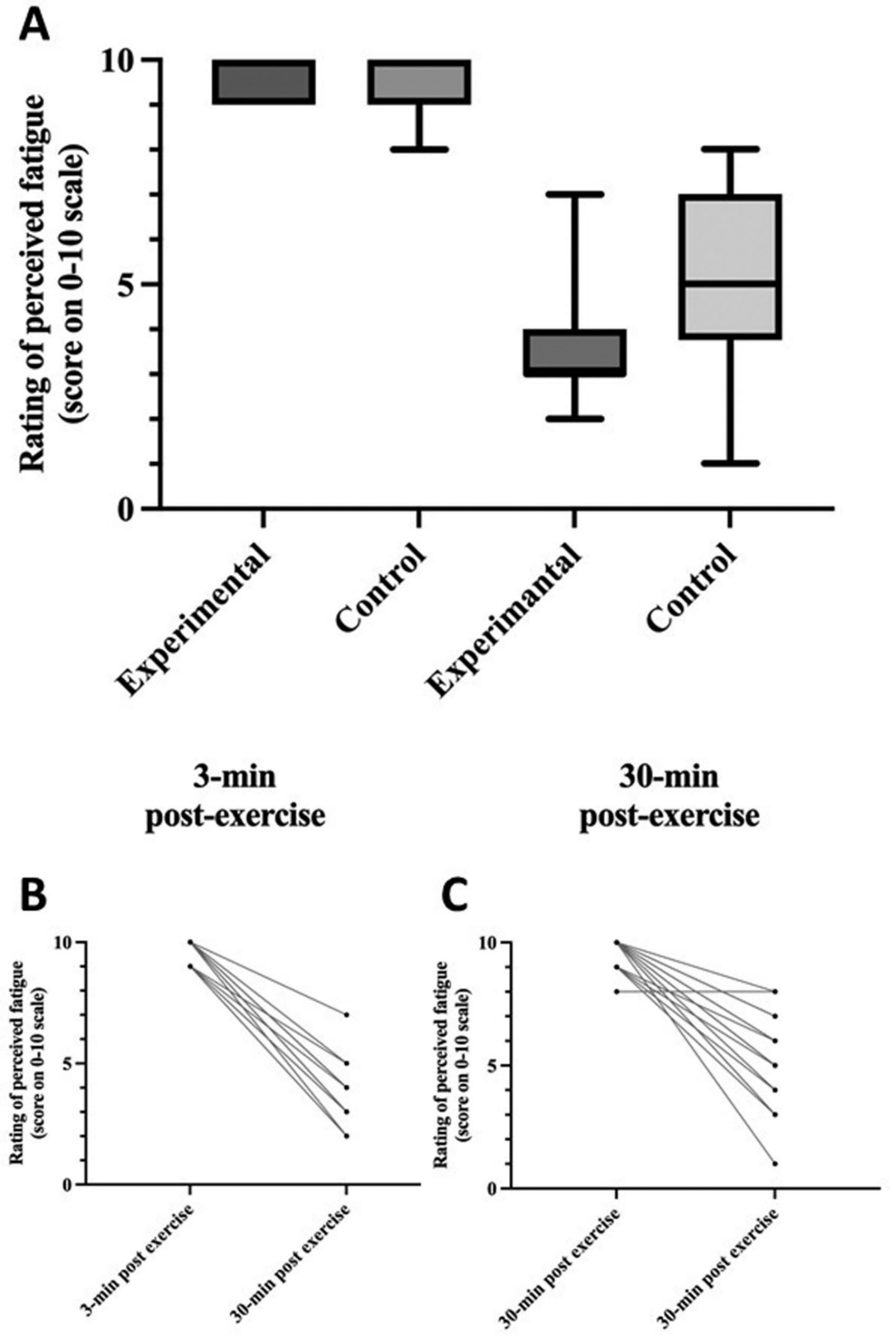
The difference in ROF between measurements at 3 min and 30 min post-exercise for both groups. Values are median and minimum or maximum values. Panel B: Individual ROF values for the experimental group. Panel C: Individual ROF values for the control group

The differences in bLa and ROF between measurements at 3 min and 30 min post-exercise are presented in Figs. 2 and 3.

Before the regression analysis, we examined the correlations between monitored variables. There was a moderate to strong correlation between S-Index Test result and maximum power (*r* = 0.74), as well as S-Index Test result and anaerobic capacity (*r* = 0.69), and between maximum power and anaerobic capacity (*r* = 0.92). Consequently, both maximum power and anaerobic capacity were excluded from the follow-up regression analysis.

Furthermore, multiple regression analysis was executed to analyse bLa at 30 min using a set of predictor variables (See Table 2). The regression model accounted for approximately 77.8% of the variance in bLa at 30 min post-intervention (R²= 0.778). The adjusted R²-value was 0.744. The overall regression was statistically significant and yielded an F-statistic value of 22.49 (*p* < 0.001). Among the predictor variables, the strongest effect has been noted for bLa at 3 min post-exercise (*p* < 0.001). The experimental group was associated with a significant difference of -1.032 mmol·L⁻¹ in bLa compared to the control group, holding all other variables constant. This effect was significant (*p* = 0.016). The VO_2_max also had a significant effect (*p* = 0.028). For each one-unit increase in VO_2_max, there was an associated decrease of 0.106 mmol·L⁻¹ in the bLa. It was also found that the S-Index Test Score was significantly related to bLa (*p* = 0.040).

**Table 2:** Results of multiple linear regression for prediction of bLa concentration at 30 min post-exercise

	Coefficient	SE	t-stat	*p*-value
**Intercept**	1.972	2.687	0.734	0.468
**Experimental Group**	-1.032	0.407	-2.537	0.016
**Sex**	0.389	0.645	0.603	0.551
**S-Index**	0.023	0.011	2.138	0.040
**VO**_**2max**_	-0.106	0.046	-2.304	0.028
**bLa concentration at 3 min post-exercise**	0.693	0.092	7.506	< 0.001

Note: Participation in the experimental group is calculated as 1 and participation in the control group is calculated as 0. Sex is calculated as 1 for males and 0 for females. VO_2max_ is calculated in mL·kg⁻¹·min⁻¹. bLa concentration is calculated in mmol·L⁻¹. Abbreviations: SE, standard error; VO_2max_, maximal oxygen uptake; bLa, blood lactate

The multiple regression analysis was again executed to analyse ROF at 30 min using a set of predictor variables (See Table 3). The regression model accounted for approximately 29.2% of the variance in ROF at 30 min post-intervention (R²= 0.292). The adjusted R²-value was 0.182. The overall regression was statistically significant and yielded an F-statistic value of 2.644 (*p* = 0.041). The experimental group was associated with a statistically significant decrease in ROF ratings (*p* = 0.006). None of the other predictor variables were statistically significant at the conventional significance level (*p* > 0.05).

**Table 3:** Results of multiple linear regression for prediction of ROF at 30 min post-exercise

	Coefficient	SE	t-stat	*p*-value
**Intercept**	4.235	6.477	0.654	0.518
**Experimental Group**	-1.560	0.531	-2.937	0.006
**Sex**	0.425	0.876	0.485	0.631
**S-Index**	0.010	0.013	0.727	0.472
**VO**_**2max**_	0.009	0.061	0.146	0.885
**ROF at 3 min post-exercise**	-0.122	0.515	-0.238	0.814

Note: Participation in the experimental group is calculated as 1 and being in the control group is calculated as 0. Participation is calculated as 1 for males and 0 for females. VO_2max_ is calculated in mL·kg⁻¹·min⁻¹ Abbreviations: SE, standard error; VO_2max_, maximal oxygen uptake; ROF, rating of perceived fatigue

## Discussion

The main objective of the present study was to investigate the influence of the low-intensity VIH protocol performed after a maximum anaerobic effort in well-trained short-track speedskaters. We found that VIH insignificantly reduced bLa in the experimental group compared to the control group (-40.8% vs. – 33.2%). We also found that VIH reduced ROF to a significantly larger extent in the control group compared to the experimental group (-63.7% vs. -46.5%). However inconclusive, our results provide preliminary evidence supporting the efficacy of VIH as a recovery protocol in high-performance settings.

Our findings regarding improvement in bLa clearance after a single VIH effort are consistent with the research from Perret et al., who reported a lack of enhanced bLa disappearance associated with VIH, when performed after exhaustive arm cranking [24]. However, his study found no significant differences between passive recovery, active recovery based on low-intensity arm cranking, and VIH. The authors speculated that the magnitude of the muscle mass involved in the physical activity was critical to efficient recovery and both respiratory and arm muscle activity did not involve enough muscle mass to elicit enhanced bLa clearance. Interestingly, the local influence and muscle engagement depend on the RMT method, possibly affecting the involved muscle mass [31, 32]. Similar conclusions were reported in the study by Johnson et al., where no association between inspiratory loading and bLa clearance was found in individuals of moderate endurance training status [30]. Noteworthy, the training status of our study participants was significantly higher, which may partially explain the difference between the studies’ results. On the other hand, Chiappa et al. reported improved post-exercise bLa clearance associated with inspiratory loading [25]. The decrease in bLa was attributed to elevated lactate uptake by the inspiratory muscles and heart [25, 31]. Interestingly, the differences in bLa between inspiratory loading and passive recovery were more apparent during the first 5 min of the recovery, indicating a faster bLa decline pattern compared to traditional protocols based on skeletal muscle engagement [25]. This may suggest that VIH recovery protocols may be particularly beneficial when there is minimal time between successive efforts. Similar findings were presented by Brown et al., who observed that loading of trained inspiratory muscles speeds lactate recovery kinetics [32].

In our study, we found a statistically significant interaction between VO_2_max and post-exercise bLa clearance. VO_2_max is associated with cardiorespiratory fitness, endurance performance potential, and health status [33, 34]. The interaction between VO_2_max and bLa clearance indicates the vital role of aerobic fitness in repeated-efforts ability in short-track speedskaters. Our finding is consistent with existing literature, which reports that high aerobic fitness improves recovery after bouts of high-intensity intermittent exercise [35, 36]. This improvement is attributed to elevated aerobic enzyme concentration and increased mitochondrial function [37], size, number, and surface area [38], as they contribute to improved oxygen extraction by the working muscles. Moreover, high aerobic fitness is associated with increased muscle blood flow, capillarization of muscle tissue, blood, and total hemoglobin volume, which improves oxygen delivery as well as lactate metabolism and transport [39]. Consequently, more energy is supplied through the aerobic and phosphagen systems with decreased contribution of anaerobic glycolysis. Furthermore, higher VO_2_max is associated with larger post-exercise oxygen uptake, resulting in faster replenishment of adenosine triphosphate and phosphocreatine [35]. Our data confirms the crucial role of aerobic conditioning in sports, where repeated high-intensity efforts are required in competition [40, 41].

Interestingly, the higher S-Index Test Score was associated with a smaller post-exercise reduction in bLa. The result stands in contrast to well-established findings about RMT improving inspiratory muscle strength and decreasing bLa simultaneously [2]. We cannot provide a mechanistic explanation for our findings. Speculatively, there may be a difference in lactate kinetics depending on respiratory muscles’ training status. As we operate in the understudied area, further research is required.

The influence of VIH on ROF values showed a significant main effect. Previously, RMT was proven to have a positive influence on the perceived rating of fatigue or exertion in multiple studies [42]. The decrease associated with respiratory training may be related to the reduction in perceived breathlessness [1], and the influence of the diaphragm activity on the autonomic nervous system, as vagal nerve stimulation may attenuate negative physiological stress reactions [18, 19]. However, cognitive reappraisal or distraction may also positively influence perceptual indices [43]. Therefore, it is possible that in our study VIH served as a mere distraction, diverting participants’ attention away from the recent discomfort of exercise towards intentional breathing and a simple, controllable task. Nevertheless, the assessment of perceptual indices is often used to predict performance [44] and is known to influence exercise capacity [20]. Traditional approaches to understanding exercise tolerance have primarily centered around the cardiovascular, respiratory, metabolic, and neuromuscular aspects of fatigue [45]. In contrast, more recent research has questioned the conventional paradigm in exercise physiology and highlighted the significant influence of the brain and subjective measures in governing exercise performance [46, 47]. Therefore, lower ROF values associated with VIH and improved temporary perceptual state might positively influence future performance, i.e. during approaching competition rounds.

The recovery protocol applied in our study was designed according to the ‘minimum effective dose’ principle, due to time constraints occurring in typical short-track competitions. In our previous study, 5 min of VIH with 20 breaths·min-1 decreased bLa in well-trained triathletes, although no high-intensity exercises were performed before VIH, and all the study participants remained in a moderate intensity domain according to RPE, bLa, and cardiac indices [23]. In the present study, the experimental group performed VIH for 3 min with 20 breaths·min-1 only, which may be described as a short and low-intensity activity. Consequently, the applied protocol may exhibit limited effectiveness. In multiple studies, effective active recovery protocols take between 20 and 30 min [10]. However, protocols of such duration may have limited applicability during short-track competition. Applying the 3-minute protocol originated from real-life constraints and exhibits a practical approach to athletes’ recovery. Since lactate clearance associated with active recovery depends on applied exercise intensity [48] and duration [49], it is likely that a longer RMT-based protocol with higher intensity or duration may elicit larger desired outcomes. Moreover, breathwork based on cyclic sighting with an emphasis on prolonged exhalations has been found especially useful in improving mood and physiological arousal [50]. Therefore, different breathwork coordination applied with VIH might be even more efficient in attenuating perceived fatigue and exertion.

Breathwork recovery protocols remain an understudied area. Our investigation not only describes part of the phenomenon but also shows the need for further research. Future investigations may include breathing protocols of different coordination, intensity and duration, monitoring a larger number of stress- and fatigue-related indices, or focusing on explaining the mechanisms behind the influence of breathwork and RMT in terms of psychophysiological recovery.

## Study strengths and limitations

The unique population of elite athletes and the expediency of the findings may be considered the study’s strengths. However, the presented research is not free from limitations. The participants were not well-familiarized with VIH. Despite the supervision of a qualified physiotherapist, the execution of the breathing protocol may have been subpar, resulting in lower diaphragm engagement. Both the WAnT and CPET were performed on the same day, which may have influenced the subsequent CPET performance. Moreover, the study participants were exclusively well-trained athletes. The group homogeneity allowed to control and minimize confounding variables, however also significantly limited the generalizability of our findings. Additionally, only a limited number of parameters were monitored during the study. Fatigue is a complex phenomenon, and the investigated indices provide only limited insight into athletes’ recovery. Therefore, further research with a larger number of monitored parameters is required to holistically evaluate VIH as a recovery protocol.

## Conclusions

In conclusion, our study has provided preliminary insights into the application of VIH as a recovery protocol in well-trained short-track speedskaters, revealing noteworthy trends. Underlying the positive effect on perceptual indices, VIH may serve as an efficient recovery protocol in high-performance settings. However, as the influence of respiratory protocols on athletes’ recovery remains an understudied area, further research to address the remaining uncertainties is required. Moreover, the significant interaction between VO_2_max and post-exercise bLa clearance was noticed, indicating the vital role of aerobic fitness in short-track speedskaters, as repeating high-intensity efforts is required in competition.

### Electronic supplementary material

Below is the link to the electronic supplementary material.

Supplementary Material 1

## Data Availability

Data will be made available upon reasonable request to the corresponding author (T.K.).
